# Performance comparison and future perspectives of deep learning and classical machine learning in bone tumor applications: a systematic review (2019–2025)

**DOI:** 10.1186/s12911-026-03401-8

**Published:** 2026-02-24

**Authors:** Yu Qiao, Carolin Eisfeld, Rüdiger von Eisenhart-Rothe, Florian Hinterwimmer

**Affiliations:** 1 Department of Orthopaedics and Sports Orthopaedics, School of Medicine and Health, TUM University Hospital, Technical University of Munich, Ismaninger Str. 22, 81675 Munich, Germany; 2 Institute for AI and Informatics in Medicine, School of Medicine and Health, TUM University Hospital, Technical University of Munich, Munich, Germany

**Keywords:** Artificial intelligence, Bone tumor, Deep learning, Classical machine learning, Diagnostic performance, Systematic review

## Abstract

**Background:**

The diagnosis and prognostic assessment of bone tumors represent a complex and clinically significant challenge. In recent years, the rise of artificial intelligence (AI), particularly deep learning (DL) and classical machine learning (ML), has emerged as a promising tool in this field. This study systematically reviews the applications of AI in bone tumor diagnosis, prognosis, segmentation, and treatment response, with a focus on model performance, emerging trends, and current limitations.

**Methods:**

This systematic review follows to the PRISMA guidelines and conducted a comprehensive search of four major databases (PubMed, Web of Science, Scopus, and Cochrane Library) to identify studies published between January 2019 and May 2025 on the application of AI in bone tumors. Relevant original articles were identified based on predefined inclusion and exclusion criteria, and research data such as basic information, algorithms, models, performance metrics, and clinical tasks, were systematically extracted and analyzed. And the performance of DL and ML methods in bone tumors was comparatively analyzed.

**Results:**

The review included 70 studies involving 53,149 cases, of which 45.83% were malignant bone tumors. DL was used in 77.63% of the studies and classical ML in 22.37%. Diagnostic tasks dominated the research focus (81.94%), followed by survival prediction (11.11%) and treatment response evaluation (6.94%). Performance metrics indicated that DL models exhibited higher weighted averages in accuracy (0.87), AUC (0.89), sensitivity (0.84), specificity (0.88), precision (0.81), and F-score (0.84), while classical ML models achieved the highest precision (0.90). Although DL demonstrated a performance advantage in image-based tasks, classical ML maintained greater stability in structured datasets. No significant performance differences were observed between large-sample and small-sample studies, reflecting the robustness of both model types. Additionally, a recent shift in research focus was observed, from diagnostic applications toward disease prediction.

**Conclusion:**

Artificial intelligence has demonstrated strong performance and potential in bone tumor research. DL often demonstrates more balanced performance in image-based bone tumor tasks, while classical ML remains competitive and may hold advantages in structured, small-sample datasets, precision-prioritized settings. However, we did not observe statistically significant differences, so these findings should be interpreted as performance tendencies in specific contexts rather than universally validated superiority. Future research should focus on optimizing DL and classical ML models, developing fusion algorithms in bone tumors can improve the generalization performance, accuracy, and ability to adapt to complex data scenarios. At the same time, fostering interdisciplinary and multicenter collaborations between computer scientists and clinicians, improving data-sharing frameworks, and addressing ethical and privacy concerns will be essential to fully harness the significant potential of AI in bone tumor research and clinical applications.

## Introduction

The incidence of primary bone tumors is 2–3 cases per 100,000 population, accounting for approximately 6.2% of all tumors [1]. Despite the overall incidence of bone and soft tissue malignance is relatively low, they account for only 0.2% of all human malignancies, they account for 10% of cancers in children under the age of 15 years and are the third leading cause of death in cancer patients under 20 years of age [2–5]. Primary bone tumors pose a uniquely difficult diagnostic challenge compared with many other solid tumors. Their imaging and histopathological appearances frequently overlap with a wide spectrum of benign, intermediate, and malignant mesenchymal and non-mesenchymal lesions. This morphological heterogeneity leads to substantial inter-observer variability, especially among less experienced clinicians, and often necessitates multi-modal evaluation combining radiography, MRI, clinical presentation, and biopsy [6]. Clinical treatment options for different types of bone tumors (benign, malignant, and intermediate) vary widely [7]. Misdiagnosis can lead to inappropriate treatment strategies, affecting limb salvage and patient survival. Therefore, early determination of the tumor’s benign or malignant nature and its staging is crucial for development of treatment strategies and assessing prognosis [8, 9]. Consequently, there is an urgent clinical need for objective, automated diagnostic, consistent, and high-quality tools to assist in this complex decision-making process.

Artificial Intelligence (AI) is a technology with human-like problem-solving capabilities that uses data and rules to make decisions and predictions through programs and algorithms [10]. Machine Learning (ML) and its subset deep learning (DL) represent different applications of AI. Where classical ML is the traditional approach in ML that relies heavily on hand-designed features and statistical modelling to learn patterns from data through algorithms, DL is the construction and training of deep neural networks (multi-layer neural networks) to allow models to automatically extract and learn complex features from data [5, 11]. Currently, the application of AI in medicine is expanding from simple data analysis to clinical practice and clinical applications [12]. Current research also concludes that the performance of AI far exceeds that of traditional analysis methods such as statistical analysis and multivariate analysis in many scenarios [13]. Significant progress has been made in the use of ML and DL for medical image analysis, enabling rapid and high-precision diagnoses [14, 15]. Moreover, AI can integrate digital pathology, clinical patient data, and clinical experience to accurately predict patient prognosis and survival [16]. The increasing application of optimization algorithms in biomedical data analysis reflects the comprehensive advancement of intelligent model optimization strategies within the healthcare research field [17]. However, as AI algorithms continue to advance and proliferate, the challenges and future research directions have become increasingly critical. These include not only ethical concerns related to AI’s interaction with human medicine but also issues surrounding the accessibility and security of large annotated patient data sets [18]. As clinical scientist, it is crucial to understand how AI can be optimally utilized in the comprehensive management of bone tumor patients. Therefore, it is essential to be aware of the current research landscape and the key areas for future advancement.

In this study, we aim to analyze and evaluate the application of AI algorithms in bone tumor research since 2019 through a systematic review. The review summarizes the primary model types, application scenarios, outcomes, and limitations while providing a comparative analysis of the strengths and weaknesses of classical ML and DL. Additionally, we discuss several unresolved issues currently encountered in clinical practice. We chose 2019 as the starting point of our review because it coincides with the launch of the European Union’s AI4EU initiative, which signified a major policy and technological push toward human-centric AI development across sectors, including healthcare.

## Materials and methods

This systematic review was conducted in accordance with the Preferred Reporting Items for Systematic Reviews and Meta-Analyses (PRISMA) guidelines [19].

### Search Strategy

In July 2025, a comprehensive search was conducted across four major databases—MEDLINE (PubMed), Web of Science, Scopus, and the Cochrane Library—targeting literature published from January 2019 to May 2025. The systematic search employed the following keywords and/or Medical Subject Headings terms, without any filters or restrictions: *(“Artificial Intelligence” OR “deep learning” OR “machine learning”) AND (“bone” OR “skeleton” OR “musculoskeletal”) AND ((malignant AND (tumor OR lesion OR neoplasm)) OR sarcoma OR malignancy).* The starting year 2019 was chosen to align with the strategic launch of the EU-funded AI4EU initiative.

### Eligibility criteria

Studies meeting the following criteria were included in this review: (1) Original articles; (2) Manuscript written in English; (3) Clinical application of AI (DL/ML, etc.). Studies were excluded if they met any of the following criteria: (1) Focus on disease-specific secondary bone tumors; (2) Soft tissue tumor; (3) Not available in full text; (4) Animal and cadaveric studies; (5) Review, case reports, letters, editorials, notes, congress abstracts, conference papers and unpublished research, etc.; (6) Significant lack of relevant evaluation data; (7) No clear source of patient information.

### Methodology of the review

Two authors (Y.Q. and F.H.) independently reviewed the collected references. Duplicate studies were removed, and the titles and abstracts were assessed based on the inclusion and exclusion criteria outlined above. For articles where eligibility could not be determined from the title and abstract alone, a full-text review was conducted to identify studies meeting the final criteria for subsequent analysis.

### Data collection

For each included study, we extracted the title, first author and country of affiliation, year of publication, data type, sample size, tumor classification (benign, malignant, intermediate), specific numbers, algorithms, models, tasks, performance metrics, outcome labels, etc. To ensure the accuracy of the final data included in the analysis, all study results were independently assessed by two reviewers, and all inconsistencies were resolved by consensus of the study team.

### Statistical analysis

For continuous parameters, the corresponding range, median, mean, standard deviation (SD), and interquartile range (IQR) were calculated. For discrete parameters, the occurrence counts and corresponding percentages of each metric were recorded. Due to significant heterogeneity in evaluation metrics, imaging data source, validation strategies, and other aspects of AI, formal meta-analysis is considered inappropriate. To present a representative overview of the field and minimize the impact of small-sample outliers, performance metrics were calculated using a sample-size-weighted average formula (Weighted $\:x$ =$\:\:\frac{{\sum\limits_{i=1}^{n}}(xi\times\:Ni)}{{\sum\limits_{i=1}^{n}}Ni}$, x represents the applied metrics in each research project, and N represents the number of cases included in the study). This ensures that studies with larger sample sizes contribute proportionally greater weight to the composite results. For performance metrics for which only interval-based data were provided in the study, we instead used the calculation of their mean values (Mean $\:x=\frac{\mathrm{M}\mathrm{i}\mathrm{n}+\mathrm{M}\mathrm{a}\mathrm{x}}{2}$).

## Result

### Results of relevant literature

**Fig. 1:**
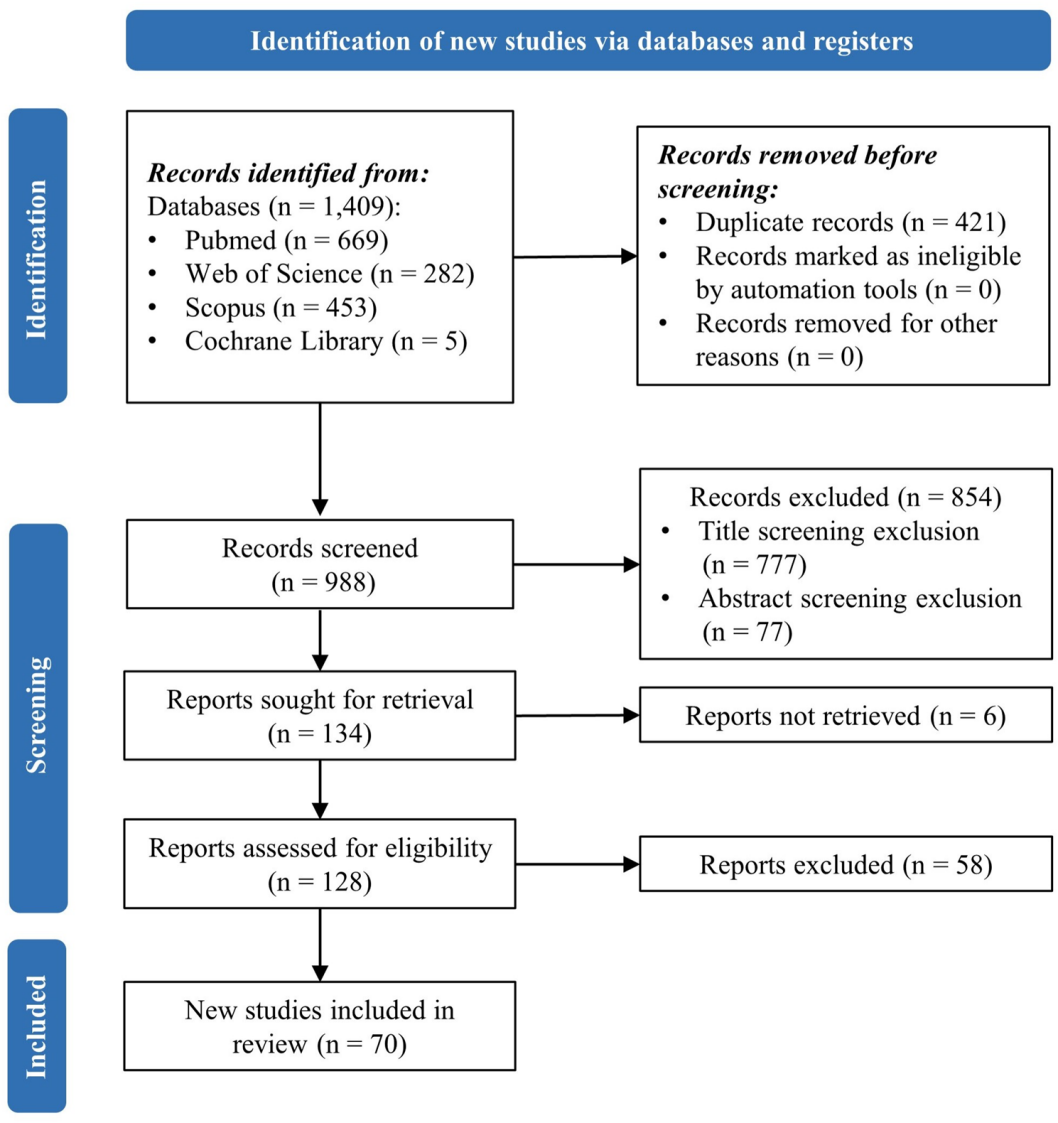
Flowchart of literature screening based on PRISMA

**Fig. 2:**
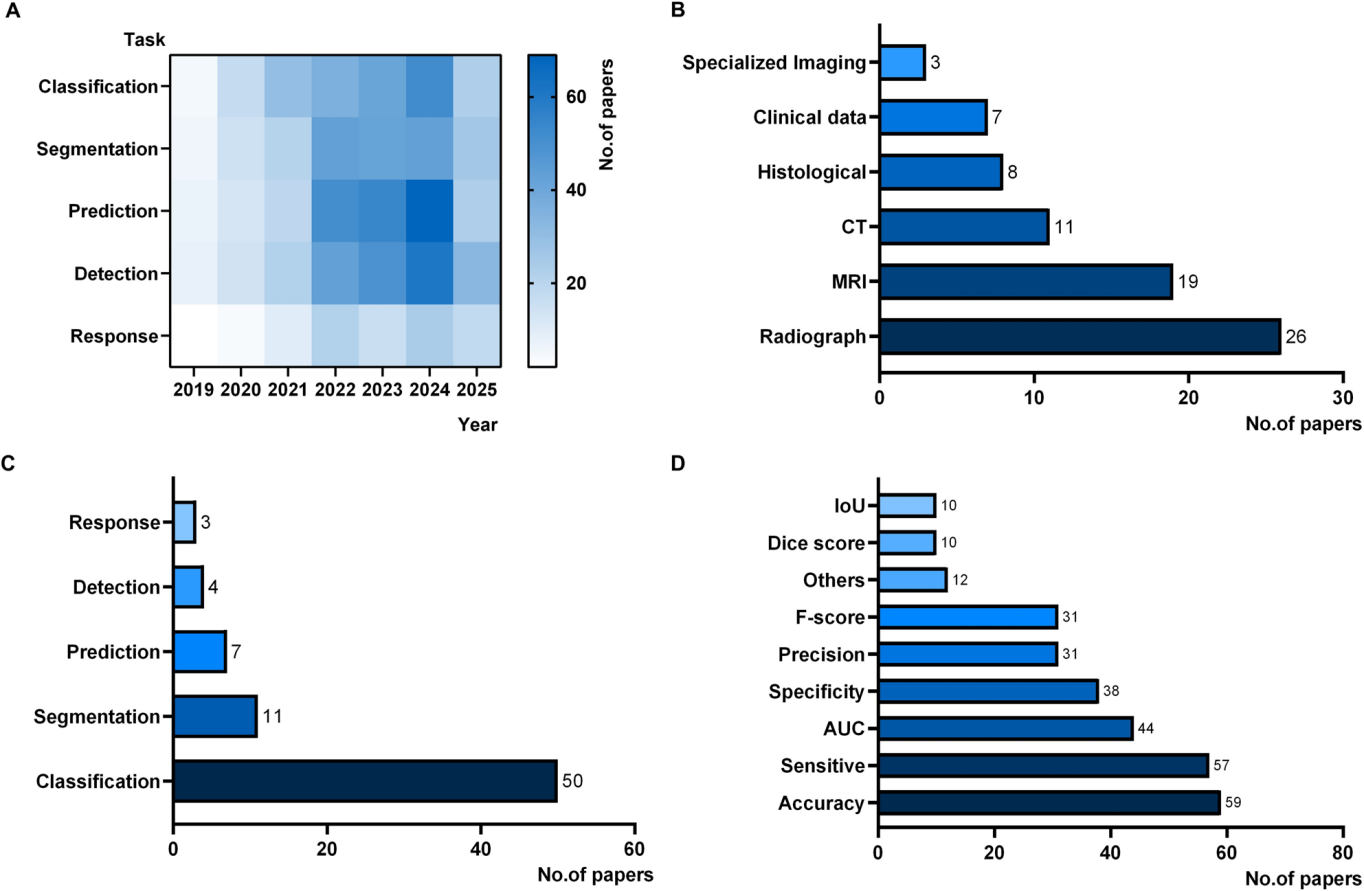
Overview of AI applications in bone tumors from 2019 to 2025. (**A**) Heatmap of research tasks across the years, showing a recent rise in prediction-oriented studies. (**B**) Distribution of imaging and non-imaging modalities, with radiograph and MRI being the most frequently used data types. (**C**) Frequency of task categories, categorization is the main focus of AI applications. (**D**) Application metrics reported across studies

Based on a search of four major databases, we identified 1409 potentially relevant records. After removing 421 duplicate studies, 988 records were screened based on their titles and abstracts, resulting in the exclusion of 860 studies. Full-text reviews were conducted for the remaining 128 studies, and 58 were excluded according to our predefined criteria. Ultimately, 70 studies met the inclusion criteria and were included in the final analysis (Fig. 1) [20–89]. Table 1 presents detailed information on the included studies, including publication details, sample types, sample sizes, research objectives, model types, and performance metrics. We conducted a heatmap analysis of all possible literature since 2019 based on the research objectives. This analysis highlights a growing focus on disease survival prediction as a research hotspot in recent years (Fig. 2A).

**Table 1:** Final article with continuous and discrete parameters and basic information

First Author	Year	National	No.Cases	Healthy Cases	Benign Cases	Intermediate Cases	Malignant Cases	Metastases Cases	Analyzing data	Purpose	Task	Model Type	Number of labels
Alabdulkreem	2023	Saudi Arabia	200	100	0	0	100	0	Radiograph	Diagnose	Classification	Deep Learning	2
Anand	2023	India	1144	536	0	0	608	0	Histological	Diagnose	Classification	Deep Learning, Classical Machine Learning	3
Breden	2023	Germany	409	220	45	0	131	0	Radiograph	Diagnose	Classification	Deep Learning	2
Chen	2024	China	500	0	335	0	165	0	MRI	Diagnose	Classification	Deep Learning	2
Cheng	2023	China	1707	0	0	0	1707	0	SEER database	Prognosis	Predicting survival	Deep Learning	Multi-class
Chianca	2021	Italy	146	0	49	0	40	57	MRI	Diagnose	Classification	Deep Learning, Classical Machine Learning	2, 3
Consalvo	2022	Germany	58	31	9	0	18	0	Radiograph	Diagnose	Classification	Deep Learning	3
Do	2021	South Korea	1576	381	1061	0	134	0	Radiograph	Diagnose	Classification	Deep Learning	3
Eweje	2021	USA	1060	0	582	0	478	0	MRI	Diagnose	Classification	Deep Learning, Classical Machine Learning	2
Georgeanu	2022	Romania	23	0	10	0	13	0	MRI	Diagnose	Classification	Deep Learning	2
Gitto	2024	Italy	150	0	0	102	48	0	Radiograph	Diagnose	Classification	Classical Machine Learning	2
Gitto	2022	Italy	30	0	0	0	30	0	MRI	Therapy	Chemotherapy response	Classical Machine Learning	2
Gitto	2020	Italy	58	0	0	0	58	0	MRI	Diagnose	Classification	Classical Machine Learning	2
Gitto	2022	Italy	158	0	0	119	39	0	MRI	Diagnose	Classification	Classical Machine Learning	2
Guo	2024	China	580	0	295	0	285	0	Radiograph	Diagnose	Classification	Deep Learning	2
Hasei	2024	Japan	1262	526	0	0	736	0	Radiograph	Diagnose	Classification	Deep Learning	2
He	2024	China	249	0	0	81	168	0	CT	Diagnose	Classification	Deep Learning	2
He	2020	China	1356	0	679	317	360	0	Radiograph	Diagnose	Classification	Deep Learning	3
He	2019	China	56	0	0	56	0	0	MRI	Prognosis	Prediction recurrence	Deep Learning	2
Hinterwimmer	2024	Germany	809	0	523	69	217	0	Radiograph	Diagnose	Classification	Deep Learning	Multi-class
Ho	2019	South Korea	963	500	329	0	134	0	Radiograph	Diagnose	Classification	Deep Learning	3
Holm	2022	Denmark	4264	0	0	0	4264	0	Clinical data	Prognosis	Predicts survival	Classical Machine Learning	2
Ibrahim	2023	The Netherlands	2365	1543	0	0	822	0	Bone scintigraphy	Diagnose	Classification	Deep Learning	2
Jiang	2021	China	835	0	0	0	835	0	SEER database	Prognosis	Predicts survival	Classical Machine Learning	2
Li	2023	China	1430	345	555	77	108	0	Radiograph	Diagnose	Classification	Deep Learning	Multi-class
Li	2022	China	1201	0	0	0	1201	0	SEER database Clinical data	Prognosis	Prediction metastasis and survival	Deep Learning	Multi-class
Liu	2022	China	585	0	270	0	315	0	MRI	Diagnose	Classification	Deep Learning	2
Liu	2022	China	643	0	392	93	158	0	Radiograph	Diagnose	Classification	Deep Learning, Classical Machine Learning	3
Liu	2021	China	3352	0	1882	0	0	1470	99mTc-MDP	Diagnose	Detection metastasis	Deep Learning	2
Magdy	2023	Egypt	581	219	0	0	362	0	Gamma camera	Diagnose	Detection metastasis	Classical Machine Learning	2
Malibari	2022	Saudi Arabia	1144	536	0	0	608	0	Histological	Diagnose	Classification	Deep Learning	3
Motohashi	2023	Japan	435	172	0	0	0	263	CT	Diagnose	Detection metastasis	Deep Learning	2
Pan	2023	China	538	324	0	0	214	0	Radiograph	Diagnose	Classification	Deep Learning	3
Park	2022	South Korea	269	60	120	0	89	0	Radiograph	Diagnose	Classification	Deep Learning	3
Saleena	2023	India	37	0	0	0	37	0	Histological	Therapy	Segment	Deep Learning	2
Sampath	2024	India	1141	511	0	0	530	0	CT	Diagnose	Classification	Deep Learning	2
Shao	2024	China	333	0	0	197	136	0	Radiograph	Diagnose	Classification	Deep Learning	2
Shuai	2023	China	23	0	0	0	23	0	CT	Diagnose	Segment	Deep Learning	2
Song	2024	China	1305	0	750	228	325	0	Radiograph, CT, MRI	Diagnose	Classification	Deep Learning	3
Tao	2021	China	458	0	206	96	156	0	Histological	Diagnose	Classification	Deep Learning	2, 3
Vezakis	2023	Greece	1144	536	0	0	608	0	Histological	Diagnose	Classification	Deep Learning	3
Vijayaraj	2024	India	220	0	110	0	110	0	MRI	Diagnose	Classification	Deep Learning	3
Schacky	2021	Germany	1045	0	743	0	302	0	Radiograph	Diagnose	Segment, Classification	Deep Learning	2
Wang	2023	China	81	0	0	0	36	45	CE-MRI	Diagnose	Segment	Deep Learning	2
Wang	2022	China	204	0	0	0	204	0	MRI	Diagnose	Segment	Deep Learning	2
Wang	2024	China	170	0	0	0	170	0	MRI	Diagnose	Segment, Classification	Deep Learning, Classical Machine Learning	2
Wu	2022	China	240	0	0	0	240	0	MRI	Diagnose	Segment	Deep Learning	2
Wu	2023	China	2164	0	0	0	2164	0	Histological	Diagnose	Segment	Deep Learning	2
Xie	2024	China	878	0	0	0	878	0	Radiograph	Diagnose	Classification	Deep Learning	Multi-class
Xu	2024	China	2675	1988	81	159	447	0	Radiograph	Diagnose	Classification	Deep Learning	2
Xu	2022	China	119	0	0	0	119	0	Radiograph	Therapy	Detection necrosis	Deep Learning	2
Ye	2024	China	749	0	207	66	125	0	MRI	Diagnose	Segment, Classification	Deep Learning, Classical Machine Learning	2, 3
Potter	2023	USA	84	0	24	0	60	0	CT	Diagnose	Segment, Classification	Deep Learning	2
Zhan	2023	China	89	0	0	0	89	0	MRI	Diagnose	Segment	Deep Learning	2
Zheng	2024	China	106	0	0	0	106	0	MRI	Therapy	Chemotherapy response	Deep Learning	2
Zhong	2022	China	144	0	0	0	144	0	MRI	Therapy	Chemotherapy response	Classical Machine Learning	2
Şimşek	2024	Turkey	1173	0	1173	0	0	0	Radiograph	Diagnose	Classification	Deep Learning	2
Dalai	2024	India	200	100	0	0	100	0	Radiograph	Diagnose	Classification	Deep Learning	2
Deng	2024	China	604	0	0	0	604	0	Clinical data	Prognosis	Predicts 1-year risk of reoperation	Classical Machine Learning	2
Deng	2024	China	150	75	25	0	50	0	Radiograph	Diagnose	Classification	Deep Learning	2
Gassert	2025	USA	344	0	124	0	220	0	CT	Diagnose	Classification	Deep Learning	2
Hasei	2024	Japan	846	378	0	0	468	0	Radiograph	Diagnose	Classification	Deep Learning	2
Hinterwimmer	2024	Germany	804	0	479	117	208	0	Radiograph	Diagnose	Classification	Deep Learning	Multi-class
Hong	2025	China	112	0	59	53	0	0	CT	Diagnose	Classification	Classical Machine Learning	2
Long	2024	China	76	0	0	0	76	0	CT	Diagnose	Classification	Classical Machine Learning	2
Nie	2024	China	211	0	0	123	88	0	CT, Clinical data	Diagnose, Prognosis	Classification, Prediction survival	Deep Learning	2
Rao	2024	India	1144	0	536	0	608	0	Histological	Diagnose	Classification	Deep Learning	2, Multi-class
Shouman	2024	Egypt	178	0	46	0	132	0	CT	Diagnose, Prognosis	Classification	Deep Learning	2, 3
Wang	2025	China	16	8	0	0	8	0	Histological	Diagnose	Classification	Deep Learning	2
Yao	2025	China	3746	1879	1525	0	342	0	Radiograph, Clinical data	Diagnose	Classification	Deep Learning	3

SEER: Surveillance, Epidemiology, and End Results

### Basic characteristics of the included literature

Tables 2 and 3 show the specific information of the continuous and discrete parameters, respectively. Our systematic review includes 70 original studies published between 2019 and May 2025, covering a total of 53,149 cases, of which 24,358 (45.83%) involve malignant bone tumor samples. The studies were published in various countries, including China, India, Italy, Germany, South Korea, USA, Japan, Saudi Arabia, Egypt, Greece, Romania, Denmark, The Netherlands, and Turkey. The studies were all retrospective. The most commonly analyzed imaging modalities were Radiograph and MRI, which accounted for 35.14% and 25.68%, respectively, followed by CT (14.86%), histological (10.81%), clinical data (9.46%), and specialized imaging (4.05%) (Fig. 2B). The purpose of the investigated studies was mainly categorized into three groups: disease diagnosis (81.94%), survival prediction (11.11%), and treatment response evaluation (6.94%). Specific research tasks included disease classification (66.67%), image segmentation (14.67%), survival prediction (9.33%), detection (5.33%), and chemotherapy response analysis (4.00%) (Fig. 2C). DL was used in 77.63% of the literature, and classical ML was used in 22.37%. Accuracy, sensitivity, AUC, specificity, precision and F-score are the top six metrics used in the literature with 20.21%, 19.52%, 15.07%, 13.01%, 10.62%, and 10.62% respectively (Fig. 2D).

**Table 2:** Continuous parameters with interval, median, mean IQR, and standard deviation

Parameter	Interval	Median	IQR	Mean	SD
Year of publication	[2019; 2025]	2023	2.00	2022.89	1.38
Number of patients/cases	[16; 4264]	458	992.50	753.11	875.25
Healthy	[0; 1988]	0	87.50	154.48	387.06
Benign	[0; 1882]	0	206.50	186.25	370.29
Intermediate	[0; 317]	0	0.00	27.51	61.14
Malignant	[0; 4264]	144	302.00	343.07	606.36
Metastases	[0; 1470]	0	0.00	30.38	180.22

IQR: interquartile range; SD: standard deviation

**Table 3:** Discrete parameters with incidence and percentage share per entity

Parameter	Entity	Σ	%
Country			
	China	36	51.43%
	India	6	8.57%
	Italy	5	7.14%
	Germany	5	7.14%
	South Korea	3	4.29%
	USA	3	4.29%
	Japan	3	4.29%
	Saudi Arabia	2	2.86%
	Egypt	2	2.86%
	Greece	1	1.43%
	Romania	1	1.43%
	Denmark	1	1.43%
	The Netherlands	1	1.43%
	Turkey	1	1.43%
Study design			
	Retrospective	70	100.00%
	Prospective	0	0.00%
Purpose			
	Diagnose	59	81.94%
	Prognosis	8	11.11%
	Therapy	5	6.94%
Task			
	Classification	50	66.67%
	Segmentation	11	14.67%
	Prediction	7	9.33%
	Detection	4	5.33%
	Response	3	4.00%
Model type			
	Deep Learning	59	77.63%
	Classical Machine Learning	17	22.37%
Outcome label			
	binary classification	51	68.00%
	triple classification	17	22.67%
	Multi-class	7	9.33%
Imaging modality			
	Radiograph	26	35.14%
	MRI	19	25.68%
	CT	11	14.86%
	Histological	8	10.81%
	Clinical data	7	9.46%
	Specialized Imaging	3	4.05%
Applied metric			
	Accuracy	59	20.21%
	Sensitive	57	19.52%
	AUC	44	15.07%
	Specificity	38	13.01%
	Precision	31	10.62%
	F-score	31	10.62%
	Dice score	10	3.42%
	IoU	10	3.42%
	Kappa	3	1.03%
	C-index	2	0.68%
	Brier score	2	0.68%
	auPRC	1	0.34%
	MCC	1	0.34%
	Jaccard Index	1	0.34%
	IBS	1	0.34%
	PPV	1	0.34%

### Results of classical machine learning in bone tumors

Seventeen studies utilized classical ML algorithms for case analysis. The mean accuracy was 0.81 with a SD of 0.10, suggesting generally high predictive accuracy across studies, although some models still exhibited lower performance (minimum value: 0.59). The mean AUC was 0.86 (SD: 0.10), indicating robust overall discriminatory power. Sensitivity averaged 0.81 with a relatively small SD of 0.11, reflecting consistent performance in identifying positive cases. Specificity had a slightly lower mean of 0.83 (SD: 0.09), but still demonstrated reliable ability to exclude negative samples. Precision maintained a high mean value of 0.89 (SD: 0.07), showing stable and accurate positive prediction performance across datasets. Additionally, the F-score, a harmonic measure combining precision and sensitivity, had a mean of 0.77 with a broader SD of 0.13, suggesting greater variability and occasional trade-offs between recall and precision (Table 4).

### Results of deep learning in bone tumors

A total of 59 studies employed DL algorithms for analysis, showing consistently high performance across key evaluation metrics. The mean accuracy was 0.86 with a SD of 0.11, demonstrating the overall reliability of DL models, though individual model performance ranged as low as 0.56. The mean AUC reached 0.89 (SD: 0.08), reflecting excellent discriminatory capacity across different datasets. Sensitivity averaged 0.85 (SD: 0.13), indicating the model’s solid ability to identify true positive cases, albeit with some inter-study variability. Specificity had a mean of 0.86 and a wider SD of 0.15, suggesting greater fluctuation in identifying negative cases. Precision remained strong with a mean of 0.89 (SD: 0.10), while the F-score, which integrates both precision and sensitivity, had a mean of 0.85 and an SD of 0.13, highlighting a well-balanced overall performance despite moderate variation across studies (Table 4).

**Table 4:** Continuous parameters with interval, median, mean IQR, and standard deviation

	Parameter	Interval	Median	IQR	Mean	SD
Classical machine learning	Accuracy	[0.59; 0.99]	0.80	0.12	0.81	0.10
	AUC	[0.63; 0.96]	0.89	0.13	0.86	0.10
	Sensitive	[0.59; 0.99]	0.84	0.15	0.81	0.11
	Specificity	[0.67; 1.00]	0.86	0.12	0.83	0.09
	Precision	[0.78; 0.97]	0.91	0.07	0.89	0.07
	F-score	[0.53; 0.93]	0.79	0.16	0.77	0.13
	Parameter	Interval	Median	IQR	Mean	SD
Deep learning	Accuracy	[0.56; 1.00]	0.89	0.15	0.86	0.11
	AUC	[0.62; 0.99]	0.88	0.11	0.89	0.08
	Sensitive	[0.34; 1.00]	0.88	0.17	0.85	0.13
	Specificity	[0.32; 1.00]	0.91	0.12	0.86	0.15
	Precision	[0.64; 1.00]	0.91	0.13	0.89	0.10
	F-score	[0.48; 1.00]	0.87	0.17	0.85	0.13

IQR: interquartile range, SD: standard deviation

### Impact of AI development timeline and sample size on model performance

**Fig. 3:**
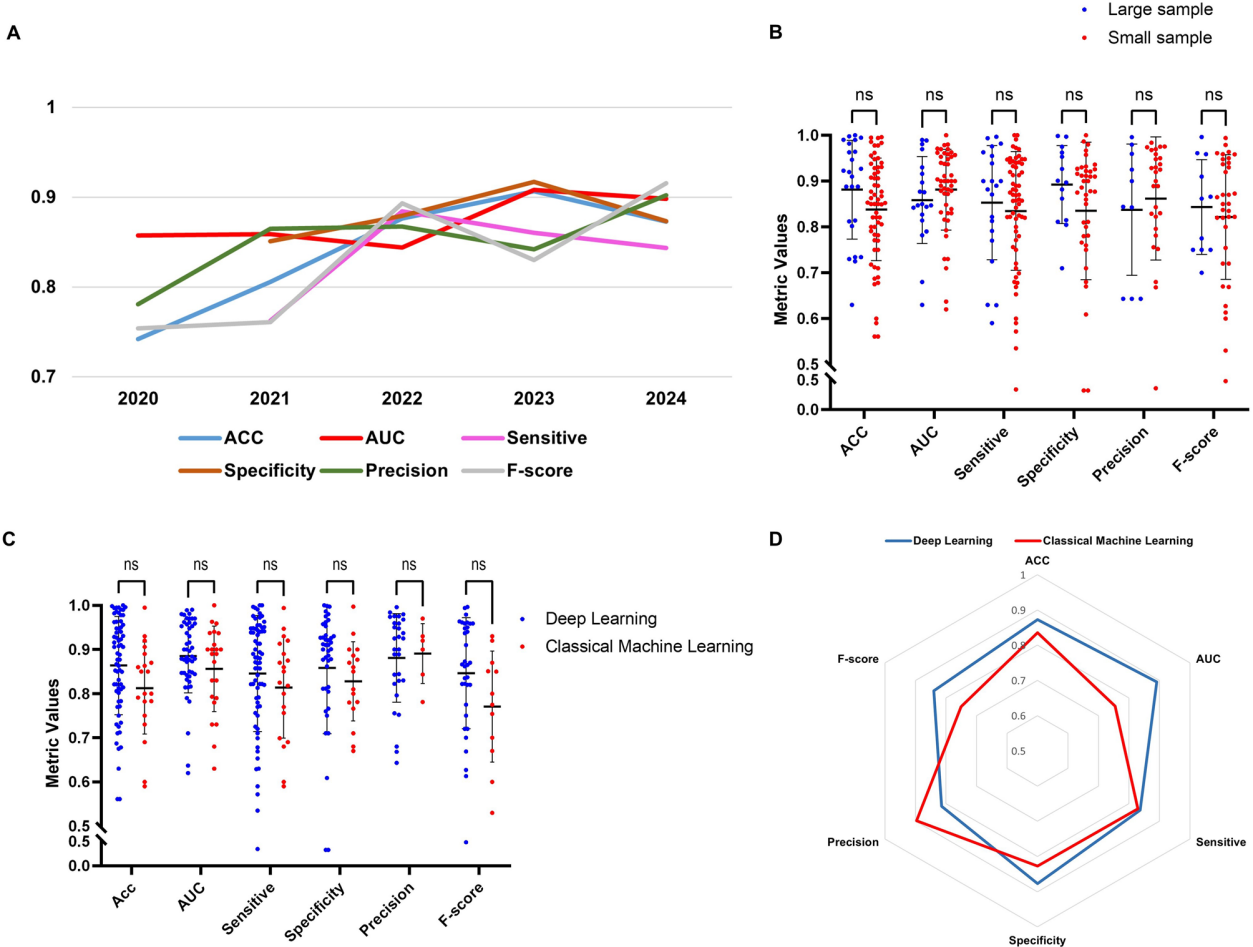
Comparative performance analysis of AI models in bone tumor studies. (**A**) Line plot of performance metrics over time (2020–2024), showing general improvements in accuracy, AUC, and specificity in recent years. (**B**) Metric distribution in relation to sample size, categorized by large (≥ 1000) and small (< 1000) sample groups. There was no statistical difference between the two groups. “Sample size” were taken as reported by the original studies and may represent either unique patients or patient-derived units (e.g., slices/patches) depending on reporting practices. (**C**) Scatterplot of various application metrics for deep learning and classical machine learning algorithms. There was no statistical difference between the two groups. (**D**) The radar plot shows the weighted of the performance metrics for both algorithms. The weighted Acc, AUC, Sensitive, Specificity, Precision, and F-score for deep learning are 0.87, 0.89, 0.84, 0.88, 0.81, and 0.84, respectively. The weighted Acc, AUC, Sensitive, Specificity, Precision, and F-score for classical machine learning are 0.83, 0.75, 0.83, 0.83, 0.90, 0.75, respectively

Figure 3A shows the temporal trend in model performance from 2020 to 2024, revealing overall improvements in all key metrics (2025 is not included as it is incomplete). Accuracy, AUC, specificity, and precision exhibited a continuous upward trend over the years. The observed performance improvements from 2020 to 2023 can be linked to concrete algorithmic advances. In particular, the increasing adoption of Vision Transformers (ViT), Swin-Transformer architectures, and attention-based feature extractors has enhanced the ability of models to capture long-range dependencies in radiographs and MRI. At the same time, self-supervised pretraining, contrastive learning, and domain-specific foundation models (e.g., RadImageNet) have significantly improved feature robustness in limited or heterogeneous datasets. These methodological developments, together with optimization strategies such as stronger data augmentation pipelines, automated hyperparameter search, and ensemble training, likely contributed to the upward trend in accuracy, AUC, and specificity observed in recent years. Notably, AUC and specificity consistently remained above 0.85 after 2021, indicating enhanced discriminatory ability and stable negative case identification. Sensitivity and F-score, however, showed more fluctuation, suggesting variability in positive case detection and trade-off balance across models. Figure 3B presents the distribution of performance metrics stratified by sample size (large sample: ≥1000 cases; small sample: <1000 cases). Despite the variation in dataset scale, no statistically significant differences were observed across all metrics (*p* > 0.05). The overlapping distributions in ACC, AUC, sensitivity, specificity, precision, and F-score suggest that sample size did not have a decisive impact on model performance in the current dataset. Although there is no statistically significant difference, this result must be interpreted with caution. One possible explanation lies in the enhanced robustness of contemporary models. An equally plausible explanation may attribute this to the “pseudo-large-sample” effect, wherein a large number of slices/tiles/patches are generated from a small patient population.

### Comparison of deep learning and classical machine learning

To compare model performance across algorithm types, we analyzed top six evaluation metrics, accuracy, AUC, sensitivity, specificity, precision, and F-score, between DL and classical ML models. As shown in Fig. 3C, DL models exhibited generally higher metric values across all parameters, though the differences between the two groups were not statistically significant (*p* > 0.05). The variability was more pronounced in the ML group, particularly for accuracy and sensitivity, suggesting less consistent performance.

Figure 3D summarizes the weighted metric values for DL and ML using radar plots. The weighted performance values for the DL model are as follows: accuracy 0.87, AUC 0.89, sensitivity 0.84, specificity 0.88, precision 0.81, and F-score 0.84. In contrast, the weighted accuracy for the classical ML group is 0.83 with an AUC of 0.75, sensitivity 0.83, and specificity 0.83, F-score of 0.75, but a precision of 0.90. These results, visualize the performance trade-off between the two algorithms. Overall, DL models demonstrated superior generalization across most metrics, particularly in AUC and specificity, which are critical for reducing false positives and ensuring diagnostic reliability. Meanwhile, ML models exhibited the highest precision (0.90), indicating strong positive predictive capability, but showed reduced consistency in global performance, as reflected by the lower F-score. These findings suggest that while DL offers more balanced and robust performance in complex tasks such as medical image interpretation, ML may still hold an advantage in structured-data scenarios or when model interpretability and precision are prioritized.

## Discussion

The complexity of bone tumors diagnosis and treatment, as well as the diversity of patient data, has become one of the most important reasons for using medical AI research. The emerging trends in artificial intelligence application for bone tumors reflect both advances in computational techniques and shifts in clinical demand. Our analysis shows a clear transition from diagnostic tasks toward survival prediction and treatment-related decision support [90]. This shift is likely driven by both deepening clinical needs and continued methodological advances. As image-based diagnostic AI has become more mature, research has moved beyond simple tumor classification toward clinically actionable objectives, including prognosis stratification, recurrence/metastasis risk estimation, survival prediction, and treatment response assessment, reflecting clinicians’ growing demand for decision support across the full care pathway [91].

Our analysis indicates that DL holds absolute dominance in bone tumors, being applied in 77.63% of studies. This is primarily attributed to DL’s suitability for processing high-dimensional image data [92, 93]. Classical ML methods, on the other hand, excel in handling structured data, particularly tabular datasets with fixed feature dimensions and clear semantics (e.g., patient clinical information). This makes classical ML particularly advantageous in tasks such as prognostic analysis [94]. Moreover, our study showed that 90.54% of the studies were based on image processing, which reasonably explains why DL is more widely used in the field of oncology, which may be related to the fact that more tumor-related studies need to be based on the processing of a large amount of image information.

Temporal analysis (Fig. 3A) indicates a steady performance improvement from 2020 to 2024. This trajectory aligns with broader advancements in AI, including the rise of Vision Transformers, Swin Transformer architectures, and foundation models, which have enhanced computer vision capabilities [95, 96]. Sample size analysis (Fig. 3B) showed no statistically significant differences in model performance between large-sample and small-sample studies across all metrics (*p* > 0.05). This observation suggests that contemporary AI models, especially DL frameworks, may have achieved sufficient robustness to maintain stable performance even in data-limited environments. However, the slightly greater dispersion of performance metrics in small-sample studies underscores the continued need for standardization in data preprocessing, annotation quality, and model validation practices to ensure generalizability. However, a more significant factor may be the reporting of “pseudo-large-sample.” Many studies included in the dataset list substantial numbers of samples, yet these samples may originate from a very small number of patients, such as multiple CT or MRI slices from the same case, different imaging sequences, or segmented pathological blocks extracted from a limited number of slides. Conversely, some studies with small sample sizes have also expanded their sample sizes through various methods. This “pseudo-large-sample” phenomenon may increase accuracy, but simultaneously obscures the authenticity of deep learning models trained on small patient cohorts [97]. This phenomenon not only obscures the true definition of sample size but also introduces the risk of “data leakage.” When studies report only the total count of images while neglecting the unique patient count, slices or patches from the same individual may inadvertently appear in both training and validation sets. Furthermore, given that classical ML exhibited superior precision (0.90) and stability in our analysis, we hypothesize that if adjusted for the actual number of patients, the advantage of ML in “true small data” regimes would be even more distinct, whereas the perceived robustness of DL in small patient cohorts might be re-evaluated.

Analysis of continuous parameters in Table 4 reveals nuanced differences in the stability and variability of model performance between classical ML and DL algorithms. For DL models, the accuracy ranged from 0.56 to 1.00, and specificity ranged from 0.32 to 1.00, indicating that although performance was often high (mean ACC = 0.86, mean specificity = 0.86), some models struggled significantly on specific datasets. Sensitivity showed the lowest minimum value (0.34), suggesting a risk of under-detection in certain clinical scenarios, though its overall mean (0.85) and median (0.88) remained high. In contrast, classical ML models demonstrated narrower ranges and slightly lower minimums: accuracy (0.59–0.99), AUC (0.63–0.96), and sensitivity (0.59–0.99). These results indicate a more consistent lower-bound performance but potentially less upward flexibility compared to DL. Notably, precision in the ML group was both high and stable (range: 0.78–0.97, mean: 0.89, IQR: 0.07), reflecting strong reliability in positive case identification. The F-score showed slightly more dispersion in the ML group (IQR = 0.16, SD = 0.13), indicating variable balance between precision and recall across studies. Importantly, for both algorithm types, the medians were close to the means across all six metrics, and IQR were generally small, particularly for precision, supporting the notion that the performance distributions were symmetric and concentrated. These findings suggest that both DL and ML models achieve relatively stable results in most studies, though DL models exhibit greater performance variability due to their broader operational range, which may reflect their adaptability to diverse data types and complexity levels.

Based on the scatter plot distribution (Fig. 3C), we observed that while DL models tended to cluster at higher performance levels with relatively low variability, the differences between the DL and classical ML groups did not reach statistical significance (*p* > 0.05). This suggests that under certain conditions, the practical performance of both approaches can be comparable. Radar chart analysis (Fig. 3D) revealed that DL models achieved higher weighted averages across most key performance metrics, particularly in AUC (0.89 vs. 0.75), specificity (0.88 vs. 0.83), and F-score (0.84 vs. 0.75). However, ML models outperformed in precision (0.90 vs. 0.81), indicating a stronger ability to correctly identify positive cases. These findings suggest that DL offers more balanced and consistent performance across a broader range of metrics, while ML demonstrates a more polarized pattern, excelling in precision but lagging in overall recall and global consistency. In summary, these results suggest that DL models are better suited for complex image-based or high-dimensional biomedical tasks due to their large parameter space and nonlinear feature extraction capabilities. In contrast, classical ML models, despite their simplicity, remain highly competitive when applied to clean, well-structured datasets, especially in diagnostic tasks where high accuracy is crucial. Currently, in addition to multicenter collaborations to expand patient samples, the development of ML algorithms for small samples is an approach that could be useful to apply to diseases such as bone tumors, which have a low incidence and prevalence [98, 99].

The DL model showed high volatility in the sensitivity index (mean of 0.85, SD of 0.13), suggesting that the ability to recognize positive samples still needs to be strengthened in model development to reduce the rate of missed diagnosis. However, deep learning performed stably in terms of accuracy (0.86), AUC (0.89) and precision (0.89), showing that it is expected to effectively reduce the burden of doctors and improve the diagnostic efficiency and consistency in the auxiliary diagnosis of bone tumors. While DL has a slight advantage in sensitivity (0.85 vs. 0.81) and specificity (0.86 vs. 0.83), classical ML exhibits lower standard deviations on several metrics, suggesting that its results are more stable. This feature makes it more applicable to small and medium sample sizes, which is of practical value especially in the context of limited data resources.

Despite high reported performance, clinical translation remains limited. The gap between research findings and clinical application is an issue that warrants attention and reflection from both researchers and clinicians. We attribute this gap to several factors. First, data heterogeneity and transparency issues persist; only 60% of studies specified tumor types, and inconsistent labeling across institutions hinders model generalizability. Second, the “Black Box” nature of DL models creates a trust gap. For high-stakes decisions, clinicians require interpretable predictions, yet many models lack transparency. Third, data privacy and ethical concerns impede the sharing of high-quality pathological data, particularly under strict regulations like General Data Protection Regulation (GDPR) [100–102]. This has led to a disconnect where computer scientists focus on algorithmic optimization without fully addressing clinical needs such as interpretability and prospective validation [5, 103].

To address the complex clinical reality of bone tumors, future research should prioritize hybrid model architectures, not merely for technical novelty, but to satisfy the unmet need for multimodal prognostic stratification [104, 105].While pure DL models excel at image perception and pure ML models dominate in structured data precision, neither can independently replicate the clinician’s workflow of synthesizing imaging phenotypes with patient clinical context. A hybrid approach is uniquely positioned to bridge this gap. This strategy justifies its computational complexity by enabling the integration of high-dimensional imaging data with low-dimensional clinical variables, thereby facilitating the transition from simple diagnostic classification to comprehensive survival prediction and treatment planning. For example, convolutional neural network (CNN) or transformer-based encoders can extract high-level imaging representations that are subsequently fed into logistic regression, support vector machines, or random forest classifiers. Meanwhile, feature selection strategies based on optimization demonstrate significant potential in cancer classification tasks [106]. Similarly, attention mechanisms can be coupled with gradient boosting models to improve feature weighting; and ensemble frameworks can merge deep feature embeddings with traditional ML decision layers to enhance stability and interpretability. Although such strategies have been successfully applied in various diseases, their integration in bone tumor research remains insufficient, opening up broad prospects for future exploration [107–110]. However, to fully unlock the potential of multimodal and hybrid systems, it is essential to acquire high-quality, diverse, and consistently annotated datasets. Achieving robust multimodal fusion necessitates overcoming practical obstacles, including heterogeneous data formats, missing modal information, and variations in imaging protocols across centers. To address these challenges while ensuring ethical compliance, future efforts should promote multicenter collaboration and adopt privacy-preserving techniques like federated learning. To address the “pseudo-large-sample” phenomenon, future medical imaging AI research should adopt a “Dual-Reporting Standard.” Researchers must explicitly distinguish and report both: the patient count (*Np*), to assess biological biodiversity and universality; and the computational sample size (*Ni*) (e.g., number of images, slices, or patches), to assess computational load and overfitting risk. This distinction is critical for systematic reviews to identify instances of performance overestimation caused by inconsistent sample size definitions. Ultimately, we should shift our focus from solely emphasizing diagnostic accuracy to developing integrated clinical decision support systems that unify diagnosis, prognosis, and treatment planning. Embedding interpretable AI modules, such as SHAP, Grad-CAM, and attention-based heatmaps, within these systems will further enhance clinicians’ trust and accelerate the practical clinical application of AI in the field of bone oncology.

Despite this systematic review included many of the literature, it still has some limitations. (i) Although we incorporated literature published up to May 2025, the rapid evolution of AI, especially the emergence of LLM, transformer, and multimodal systems, means that publication timelines may not fully capture the most recent methodological advances. (ii) This review focused exclusively on primary bone tumors and excluded secondary bone tumors. While metastatic bone disease constitutes a substantial clinical burden, secondary tumors arise from biologically heterogeneous primary cancers and exhibit diverse imaging phenotypes. Including them would introduce major variability in disease behavior, diagnostic pathways, and data distribution, thereby increasing noise and reducing comparability among AI models. In the future, we propose conducting separate, dedicated systematic reviews specifically for secondary bone metastases. (iii) Although the “black box” nature of AI models was discussed, our review could not provide a quantitative assessment of model interpretability because most included studies did not report explainability metrics. Nonetheless, methods such as SHAP, LIME, Grad-CAM, and attention-based visualization tools represent valuable strategies for enhancing transparency and clinician trust. (iv) Due to the uneven amount of research on ML and DL, coupled with inconsistencies in “sample size” reporting within the original literature (some studies failed to distinguish between patient counts and image counts). Therefore, when conducting stratified analysis, we cannot entirely eliminate the confounding effects introduced by “pseudo-large samples.” The weighting algorithm we used does not necessarily truly reflect the level between the two, but only removes the impact of the data volume bias as much as possible, and we also simply perform an inductive analysis, which could potentially influence our assessment of the model’s performance across different data scales.

## Conclusion

This systematic review highlights the potential of AI algorithms in the diagnosis, detection, survival prediction, image segmentation, and chemotherapy response analysis of bone tumors. Although statistical analysis indicates comparable overall performance between the two approaches, with both classical ML and DL methods demonstrating high accuracy across multiple tasks, DL is particularly well-suited for processing complex, high-dimensional medical images. Conversely, classical ML excels in structured data tasks with superior precision and stability. Therefore, the future direction of AI for bone tumors may not lie in choosing one over the other, but in leveraging their complementary strengths: applying DL to perception tasks while utilizing ML for decision logic. However, challenges such as small sample sizes, lack of multicenter studies, inconsistent data labeling, and the “black box” nature of AI models have hindered their clinical application. Future research should focus on developing AI models that integrate multimodal data, improve interpretability, and enhance robustness while fostering closer collaboration between computational scientists and clinicians. Addressing these issues is critical to bridging the gap between research results and clinical implementation, ultimately leading to more personalized clinical treatments and more effective clinical decision-making.

## Data Availability

Data extracted from included studies are presented in tables. All included data can also be accessed directly from the relevant publications [20, 21, 22, 23, 24, 25, 26, 27, 28, 29, 30, 31, 32, 33, 34, 35, 36, 37, 38, 39, 40, 41, 42, 43 , 44, 45, 46, 47, 48, 49, 50, 51, 52, 53, 54, 55, 56, 57, 58, 59, 60, 61, 62, 63, 64, 65, 66, 67, 68, 69].

## Abbreviations

AI: Artificial Intelligence
ML: Machine Learning
DL: Deep Learning
LLM: Large Language Model
AUC: Area Under the Curve
ACC: Accuracy
SD: Standard Deviation
IQR: Interquartile Range
PRISMA: Preferred Reporting Items for Systematic Reviews and Meta-Analyses
